# Psychometric evaluation of the Depression, Anxiety and Stress Scale (DASS-21) in peruvian university students

**DOI:** 10.17843/rpmesp.2025.423.14209

**Published:** 2025-09-29

**Authors:** Ingrid Cirilo-Acero, Marivel Aguirre-Morales, Josué Michael Franco-Mendoza, Lizley Janne Tantalean-Terrones, José Livia-Segovia

**Affiliations:** 1 Facultad de Psicología, Universidad Nacional Federico Villarreal, Lima, Peru. Universidad Nacional Federico Villarreal Facultad de Psicología Universidad Nacional Federico Villarreal Lima Peru

**Keywords:** Validation Studies, Psychometrics, University student, Anxiety, Depresión, Psychological Stress

## Abstract

**Objectives.:**

To evaluate the psychometric characteristics of the Depression, Anxiety, and Stress Scale (DASS-21) in university students from Lima, Peru.

**Materials and methods.:**

An instrumental study was conducted with 1163 students from a public university, in which the validity and reliability of the DASS-21 were evaluated in accordance with international standards.

**Results.:**

A confirmatory factor analysis was performed with a first three-factor model (χ²=706.5; df=186; CFI=0.986; TLI=0.985; RMSEA=0.049; SRMR=0.057) and a second-order model (χ²=706.5; df=186; CFI=0.986; TLI=0.985; RMSEA=0.049; SRMR=0.057), both of which reported expected fit measures. Furthermore, associations with other measures were established, such as positive mental health (r > -0.49), with results showing inverse and statistically significant moderate correlations. Metric invariance measures based on sex were also calculated. The reliability coefficients—omega, ordinal alpha, and Guttman—reached values greater than 0.90 as expected.

**Conclusions.:**

The DASS-21 showed evidence of validity based on internal structure, concurrent and discriminant criterion validity, and reliability through internal consistency. It was also invariant across men and women.

## INTRODUCTION

Epidemiological studies on mental health in Peru over the last two decades have highlighted the challenging reality of depression, anxiety, and stress. In Metropolitan Lima, two studies conducted in 2002 and 2012 reported that the lifetime prevalence of mental health disorders was 18.2% and 17.3% for depressive episodes, and 9.9% and 3% for generalized anxiety disorder, respectively ^1^^,^^2^. In 2018, hospitals and health centers recorded, in adults aged 18 to 65, a lifetime prevalence of 30.3% for depressive disorders, 7.9% for anxiety disorders, and 3.6% for post-traumatic stress disorder, with a higher frequency in women than in men ^3^.

The COVID-19 pandemic had a considerable epidemiological impact on mental health worldwide, with an emphasis on anxiety disorders, depression, and post-traumatic stress symptoms. Those conditions were identified as the most frequent problems in individuals infected with the disease ^4^. In the Chinese population, a moderate to severe psychological impact was reported, with a prevalence of 16.5% for depressive symptoms, 28.8% for anxiety, and 8.1% for stress ^5^.

Likewise, a greater presence of psychological distress has been evidenced in women, as well as higher stress scores in people aged 18 to 30 and in those over 60 ^6^. In Peru, medical personnel in the Arequipa region presented symptoms of depression in 56.7% and anxiety in 35.7% ^7^. Similarly, in a study of 64,493 Peruvian residents, 35% reported symptoms associated with major depressive episodes; furthermore, 59% of these participants reported a previous diagnosis related to a mental health disorder ^8^.

Therefore, it is a priority to investigate the utility of instruments that evaluate the aforementioned psychopathological and psychosocial aspects, which the literature describes as differentiated constructs for depression ^9^^-^^12^, anxiety ^13^, and stress ^14^, in order to promote methodological rigor and have updated measures available.

In this context, the Depression, Anxiety, and Stress Scales (DASS) emerged, developed in Australia to evaluate three related negative emotional states: depression, anxiety, and stress. Subsequently, a 21-item short version was designed ^15^^,^^16^, whose main advantage lies in its brevity and ease of application as a self-report ^17^. From an empirical point of view, depression is characterized by sadness, decreased self-esteem, and loss of transcendent goals; anxiety is associated with situational fear responses and physical symptoms; while stress is defined as a “state of persistent arousal and tension with a low threshold for feeling upset or frustrated” ^15^.

The Depression, Anxiety and Stress Scale (DASS-21) has moderate-quality evidence for content validity, high-quality evidence for bifactorial structure validity, and high-quality evidence for internal consistency. In criterion validity, only the depression subscale shows sufficient evidence of its high quality. The measure of invariance between genders shows inconsistent evidence of moderate quality; however, not enough evidence is presented to indicate a low quality of reliability of the subscales. These data correspond to a systematic review of 48 psychometric studies of the instrument ^18^.

In the international context, the DASS-21 has been translated into several languages and subjected to psychometric analyses that strengthen the three-factor structural model. This is the case of its evaluation in health professionals of Persian origin, with scores (χ²/(df) = 1457/(186); p<0.001; RMSEA=0.078; TLI=0.906; CFI=0.917; SRMR=0.047) with test-retest reliability (0.740-0.881; p<0.01) ^19^. Additionally, a three-factor construct validity of 0.837 to 0.863 was observed in male patients of Malaysian nationality ^20^.

However, in a cross-cultural study of university students from Brazil, Canada, Hong Kong, Romania, Taiwan, Turkey, the United Arab Emirates, and the United States, the bifactorial model was confirmed, including the three-factor structure (depression, anxiety, and stress) and a single factor (general distress) ^21^. These three model possibilities are replicated in Swedish university students and patients with reliability from 0.77 to 0.91 for the depression, anxiety, and stress dimensions ^22^. On the other hand, in an adaptation of the Hindi language for patients diagnosed with cancer, a four-factor model was evaluated; however, the three-factor model showed greater internal consistency with an alpha value of>0.990 ^23^.

In Peru, the DASS-21 has demonstrated psychometric evidence in adolescents in regular basic education, with an adequate factorial fit and alpha and omega coefficients above 0.80, as well as invariance by sex and age ^24^. In the university population, both the tripartite factorial division and the unidimensional one were replicated, although specific factors of a residual nature were identified ^25^. In users of a community mental health center in Lima, a three-factor structure was confirmed in 12 items, with omega reliability coefficients of 0.83 for depression, 0.73 for anxiety, and 0.71 for stress ^26^.

The review of the instrument’s background highlights the need for standardized tools for use in clinical and scientific practice, which enable an accurate diagnosis of users, the evaluation of intervention effectiveness, and the development of high-impact epidemiological studies ^17^. In this sense, the psychometric evidence of the DASS-21 in Peru requires further exploration in a university population with the largest possible sample size, as suggested in previous studies ^25^. Likewise, it is relevant to analyze the structural properties of the instrument to determine if they are maintained between men and women, in order to assess its discriminative capacity, as it is a clinical tool.

Therefore, the objective of this research was to evaluate the psychometric characteristics of the DASS-21 in university students from a public institution in Lima. The specific objectives were to identify the internal structure, evaluate the evidence of criterion validity, and analyze the internal consistency of the scores.

**KEY MESSAGES**

Motivation for the study. In Peru, mental health problems continue to rise, highlighting the need for an instrument with validity and reliability standards adapted to the Peruvian university context to assess the constructs of depression, anxiety, and stress.

Main findings. The validity of the internal structure, concurrent validity, and reliability of the instrument were demonstrated in a university population.

Implications for public health. The demonstration of psychometric evidence supports the continuation of future studies aimed at establishing the prevalence of depression, anxiety, and stress; additionally, it allows its use as a screening test in the mental health field.

## MATERIALS AND METHODS

### Design

The research was framed within an instrumental design ^27^, as the DASS-21 was evaluated in terms of validity and reliability, in accordance with international standards.

### Population and sample

The study included 1163 of the 14,616 students enrolled at the Universidad Nacional Federico Villarreal (Lima, Peru). The sample size, calculated considering a 3% margin of error and a proportion variability of 50% (p=0.5; q=0.5) ^28^, required at least 995 participants for a 95% confidence level. However, anticipating rejections and losses, 1163 students were included.

### Instruments

The DASS-21 is a short version of the original instrument developed by Lovibond and Lovibond ^15^. It evaluates the absence, presence, and intensity of subjective and specific emotional symptoms associated with depression and anxiety; in the case of stress, it assesses activation in the face of persistent behavioral difficulties. Each scale consists of seven items, scored from 0 to 3, resulting in a maximum score of 21 per dimension. Its application focuses on a recent time period. The instrument was originally developed in English and adapted to Spanish starting in 2002 ^29^.

The Warwick-Edinburgh Mental Well-being Scale, developed by Tennant et al. ^30^, consists of 14 positively focused items related to emotional and functional well-being, each with five response alternatives that assess experiences from the previous two weeks. Although initially designed to monitor participants in a positive mental health promotion program, subsequent research highlights its utility for detecting clinically significant changes. This instrument has psychometric evidence in both university and general populations ^30^. It presents a unifactorial structure with high internal consistency and adequate reliability indicators ^31^, and has also been tested in a Latin American context ^32^. With this background, its psychometric properties were evaluated in a sample similar to that of the present study, confirming its unifactorial structure with adequate fit indices (CMIN=2.196; GFI=0.997; CFI=0.998; NNFI=0.998; RMSEA=0.043; SRMR=0.031), and an internal consistency of McDonald’s omega and ordinal alpha of 0.826 and 0.799, respectively.

### Procedure and data analysis

Data collection was conducted using a virtual Google Form, which included information about the study’s objectives, the assessment instruments, the minimum age requirement (18 years), the scope of participation, and a guarantee of confidentiality. The form contained an informed consent section; those who accepted it accessed questions about sex, age, year of study, and 45 items corresponding to the selected scales. The average response time was 10 to 15 minutes.

Recruitment was conducted by sending the form to the institutional email of currently enrolled students and by publishing it on the university’s official Facebook page. After 15 days, given the need to increase participation, dissemination was extended to student WhatsApp groups. The collection phase lasted 60 days. After the process, the responses were downloaded to an Excel file and subsequently analyzed with the JASP (version 0.16.1) and R (version 4.1.2) statistical packages.

The psychometric evaluation included the calculation of Pearson product-moment correlation coefficients to estimate the discriminative capacity of the items ^33^ and the relationships between dimensions ^34^ using heterotrait-monotrait ratio (HTMT) indices ^35^. Likewise, a confirmatory factor analysis (CFA) was performed to identify the instrument’s dimensions and calculate McDonald’s omega and ordinal alpha coefficients to estimate the internal consistency of the scores. This process was carried out after cleaning potential outliers that could generate measurement bias.

Estimates from Diagonally Weighted Least Squares ^36^ were also used, as it is the most robust model against heteroscedasticity, supported by features that improve estimator robustness and better manage error variability by working with the diagonalization of the variance-covariance matrix. In this sense, the analysis was conducted using the EQS emulation of the free statistical package JASP, version 0.14.1.0, with a 99% confidence interval and a 5% estimation error.

The process involved estimating the fit parameters of two possible measurement models for the DASS-21: a) an independent three-factor and a second-order factor model, and b) a related three-factor model, taking into consideration the versatility that the structural and measurement model can present with nomological and discriminant bases between scales ^37^^,^^38^.

A minimum criterion of a goodness-of-fit index (GFI)>0.95, comparative indices (CFI, TLI), and non-normalized comparative indices (NNFI) ^39^ with minimum values above 0.90, and expected error indices below 0.08 were considered for both the root mean square error of approximation (RMSEA) and the standardized root mean square residual (SRMR). The path diagram was used with structural equation modeling (SEM), expecting factor loadings greater than 0.30 ^36^, as well as the variability provided by the item, factor, and the regression and covariance measures between latent variables. Concurrent validity was also analyzed by contrasting the DASS-21 factors with the Warwick-Edinburgh Mental Well-being Scale from a divergent perspective. The coefficient values were considered large (>0.70), moderate (>0.50), or small (>0.30) ^40^. Finally, reliability was estimated using McDonald’s omega, ordinal alpha, and Guttman’s split-half, with reference scores >0.70 and item-test correlations >0.30 for each element in each factor ^41^.

### Ethical considerations

The study followed the norms of the Ethics Committee of the Research, Innovation, and Entrepreneurship Unit of the Faculty of Psychology, which was expressed with the approval of Opinion N°003, in accordance with RR.N°077-2022-UNFV of the Universidad Nacional Federico Villarreal, the principles established by the College of Psychologists of Peru, the American Psychological Association, and the Declaration of Helsinki, including only those participants who agreed to be part of the study by giving their consent in the informed consent form.

## RESULTS

Of the total participants, 30.5% (355) were men and 69.4% (808) were women. The students’ age ranged from 18 to 32 years, with an average of 25 years and a standard deviation of 3.72.

### Evidence of validity from confirmatory factor analysis

The analysis of the DASS-21 Scale’s measurement model was carried out, considering the conformation of three related factors and three independent factors with a second-order factor. The contrast of the fit measures against the representative scores by the mean was performed with the robust DWLS (Diagonally Weighted Least Squares) estimation in the EQS emulation. The results of the analysis in these two models are shown in Table 1.

**Table 1:** Fit indices for the Depression, Anxiety, and Stress Scale (DASS-21).

Model	Adjustment				Comparative			Residual	
	x^2^	gl	p	GFI	CFI	NNFI	TLI	RMSEA	SRMR
Three factors	706,5	186	1,00	0,987	0,986	0,985	0,985	0,049	0,059
Second order	706,5	186	1,00	0,987	0,986	0,985	0,985	0,049	0,059

χ²: Chi-square, df: degrees of freedom, p: significance level, GFI: goodness-of-fit index, CFI: comparative fit index, NNFI: non-normed fit index, TLI: Tucker-Lewis Index, RMSEA: root mean square error of approximation, SRMR: standardized root mean square residual

**Figure 1:**
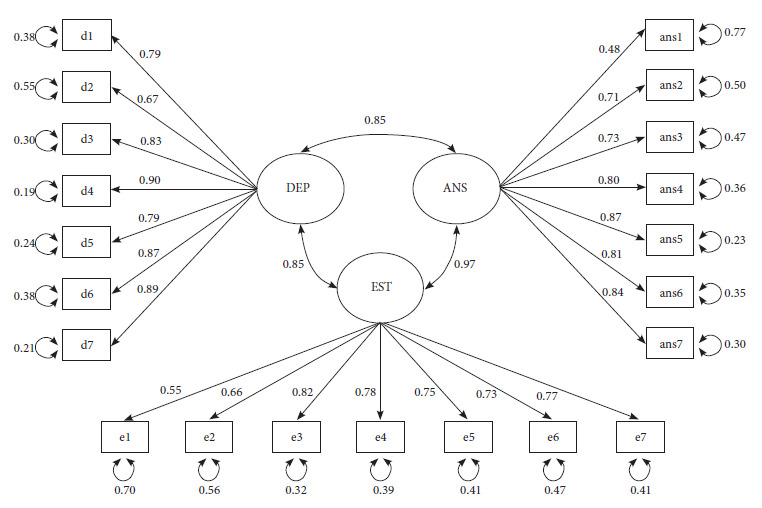
Path diagram representing the first-order model of the DASS-21 with three related factors.

It is observed that the measures obtained an adequate fit in both models, being similar and exceeding the minimum established standards (χ²=706.5; df=186; p<0.001; GFI=0.996; CFI=0.986; NNFI=0.985; TLI=0.985; RMSEA=0.049; SRMR=0.057). Graphically, the model is represented in Figure 1.

EST/e: stress; ANS/ans: anxiety; DEP/d: depression

Likewise, it should be noted that parametric invariance measures based on sex were obtained. The variations between the comparative and residual fit indices according to the invariance criteria were less than 0.01, which can be observed in Table 2.

**Table 2:** Invariance measures by sex for the three-factor model.

Measures	x^2^	CFI	△CFI	RMSEA	△RMSEA	SRMR	△SRMR	p
Model	706,500	0,986		0,049		0,057		< 0,001
Configural	830,119	0,988	0,002	0,046	-0,003	0,063	0,006	< 0,001
Metric	904,170	0,986	0,000	0,048	-0,001	0,066	0,009	< 0,001
Scalar	939,187	0,988	0,002	0,047	-0,002	0,064	0,007	< 0,001
Strict	977,990	0,985	-0,001	0,047	-0,002	0,066	0,009	< 0,001

χ²: Chi-square, p: significance level, CFI: comparative fit index, RMSEA: root mean square error of approximation, SRMR: standardized root mean square residual, △: variation

The factor loadings obtained by the model’s conformation remained oscillating between 0.43 and 0.98 in all items with the related factors model, respecting the original conformation of the test. The covariance measures between factors were 0.846 between depression and anxiety; 0.877 between depression and stress, and 0.984 between anxiety and stress. Thus, the conformation of the DASS-21 measurement model with the first-order model and its respective latent variables was confirmed, ratifying the original proposal suggested in the literature regarding the scale, as observed from Table 3.

**Table 3:** Factor loadings and latent variable regressions of the model.

Factor	Ítem	Estimate	EE	z	p	95% CI		Loading
						Lower	Upper	
EST	es1	1,000	0,000			1,000	1,000	0,481
	es2	1,227	0,084	14,691	< 0,001	1,064	1,391	0,622
	es3	1,584	0,099	16,054	< 0,001	1,391	1,778	0,763
	es4	1,289	0,087	14,802	< 0,001	1,118	1,460	0,666
	es5	1,550	0,081	19,076	< 0,001	1,391	1,710	0,716
	es6	1,279	0,082	15,600	< 0,001	1,118	1,440	0,669
	es7	1,580	0,098	16,071	< 0,001	1,387	1,772	0,716
ANS	ans1	1,000	0,000			1,000	1,000	0,433
	ans2	1,157	0,086	13,409	< 0,001	0,988	1,326	0,591
	ans3	1,332	0,100	13,308	< 0,001	1,136	1,528	0,640
	ans4	1,679	0,119	14,057	< 0,001	1,445	1,913	0,741
	ans5	1,774	0,128	13,892	< 0,001	1,524	2,025	0,804
	ans6	1,603	0,125	12,819	< 0,001	1,358	1,848	0,713
	ans7	1,729	0,127	13,572	< 0,001	1,479	1,978	0,769
DEP	d1	1,000	0,000			1,000	1,000	0,743
	d2	0,851	0,040	21,225	< 0,001	0,772	0,930	0,597
	d3	0,885	0,042	21,294	< 0,001	0,804	0,967	0,698
	d4	1,195	0,046	26,238	< 0,001	1,106	1,284	0,853
	d5	1,012	0,041	24,467	< 0,001	0,931	1,093	0,732
	d6	1,044	0,048	21,895	< 0,001	0,950	1,137	0,768
	d7	0,947	0,044	21,773	< 0,001	0,862	1,033	0,728
DEP	ANS	0,846	0,015	57,373	< 0,001	0,817	0,875	0,846
DEP	EST	0,848	0,015	58,126	< 0,001	0,820	0,877	0,848
ANS	DEP	0,967	0,009	110,903	< 0,001	0,950	0,984	0,967

EST/es: stress, ANS/ans: anxiety, DEP/d: depression, DAS: DASS-21, SE: standard error, z: z-score estimate, p: significance level, CI: confidence interval, Lower: lower bound, Upper: upper bound.

The evaluation of divergent validity showed that positive mental health had moderate relationships with anxiety (-0.488), depression (-0.607), and stress (-0.525). Regarding discriminant validity, using the HTMT (35), it met the established thresholds, below 0.85-0.90 (according to the conservative criterion) suggests good discriminant validity, while values close to or above 1 indicate a lack of distinction between the constructs. However, the relationship between anxiety and depression (0.852) was at the limit of the discriminant validity criterion, while anxiety and stress (0.979) exceeded the 0.90 threshold, indicating a possible lack of differentiation between these constructs. Nevertheless, the relationship between depression and stress (0.852) was at the upper acceptable limit, as can be seen in Table 4.

**Table 4:** Concurrent validity by heterotrait-monotrait (HTMT) correlations

	Anxiety	Depression	Stress
Anxiety	1,000		
Depression	0,852	1,000	
Stress	0,979	0,852	1,000

Regarding the analysis of the average variance extracted (AVE), it is observed that all values are above 0.50, which indicates convergent validity (Table 5).

**Table 5:** Evidence of reliability by internal consistency and average variance extracted (AVE) for the Depression, Anxiety, and Stress Scale (DASS-21

Scale	N° of items	Omega	Alfa	Guttman	AVE
Total Scale	21	0,943	0,942	0,944	-
Stress	7	0,849	0,845	0,848	0,533
Anxiety	7	0,855	0,855	0,856	0,573
Depression	7	0,891	0,892	0,894	0,676

AVE: average variance extracted

### Reliability measures

The internal consistency coefficients for the DASS-21 scales reflected values greater than 0.80 for the omega, alpha, and Guttman statistics, which demonstrate reliability in the measures obtained by the scale. The results of the process can be seen in Table 5, where measures between 0.84 and 0.94 are observed.

The results of the analysis of the DASS-21 Scale had evidence of validity and reliability for the use of the scale in the referred population, thus confirming both the measurement model and the structure of the scale with the 21 items that formed the original proposal in the literature regarding it.

## DISCUSSION

The DASS-21 has a backing of psychometric evidence, both nationally and internationally, that confirms its structure in various contexts ^24^^,^^26^. Within this framework, the present research aimed to verify the psychometric characteristics of the DASS-21 in a sample of university students from Lima.

Through CFA, the validity of the three-factor model of the DASS-21 was corroborated, as well as the relevance of the second-order model, with fit indices consistent with those reported in the original version (29). In parallel, the unidimensional structure of the instrument and the strength of the depression, anxiety, and stress variables as independent agents are supported in Swedish ^22^, Peruvian ^25^, and participants from Brazil, Canada, Hong Kong, Romania, Taiwan, Turkey, United Arab Emirates, and the United States ^21^.

Various studies have shown the adequate quality of the items’ functioning ^19^^,^^23^^,^^42^. It has also been demonstrated that the models are invariant with respect to sex, which supports their fairness in measurement between men and women, as already observed in the adolescent population ^24^.

Regarding the factor loadings of the items on each scale of the DASS-21, it was observed that the anxiety and depression dimensions reached values close to 0.70, which reflects the contribution of each element in explaining the variability of its clinical indicators ^36^^,^^39^. In the same sense, the factor loadings that measure stress presented, on average, lower magnitudes. They have an important presence in the three-factor model and in the second-order model.

Regarding the model fit, as evidence of internal structure validity through CFA, it is necessary to contrast the advantages of the original model against the unifactorial model of “general distress” ^25^ with theoretical implications that allow the grouping of anxiogenic, depressive, and stress indicators, outside the perspective of Lovibond and Lovibond based on each construct ^16^.

In the same perspective, there are studies with similar sample sizes that do not coincide with the results of the present investigation by not demonstrating the three-dimensional structure of the scale ^43^, as has been mostly achieved in similar populations and age groups. This is attributed to the cultural and symbolic differences in the language of the scale’s versions since its first adaptation to Spanish ^29^.

To demonstrate another form of validity evidence, the association with the Positive Mental Health Scale was analyzed. The results showed inverse correlations, which indicates that the DASS-21 scores on depression, anxiety, and stress correspond to a different construct than that evaluated by said scale. Furthermore, the correlations were moderate and significant in all elements of contrast. Therefore, the DASS-21 measures showed the expected concurrent criterion validity according to the study’s hypothesis.

Regarding reliability by internal consistency, it is possible to affirm that the DASS-21 measures reached optimal values greater than 0.70 on multiple occasions, which is recommended for indicators derived from the analysis based on the alpha and omega coefficients. In the same sense, the quality that the items maintain at the grouping level is demonstrated, despite the four-point ordinality, including zero as a response option within each of the scales; an issue to which consistency problems have commonly been associated due to the variance contribution of the elements when constituting the factor. These results are comparable with the indicators of 0.77 to 0.92, considering the three-factor model of the instrument in health personnel ^22^.

One of the limitations of the present study was the use of non-probabilistic sampling. However, the applied sample size is acceptable to evidence the validity of the instrument ^44^^,^^45^. The results cannot be generalized, as the participants came from a single institution. Furthermore, being a cross-sectional study, it was not possible to estimate temporal stability through test-retest. There were also no clinical samples, which prevented the analysis of discriminant validity and the estimation of specificity and sensitivity using ROC curves, procedures necessary to support its diagnostic application.

Likewise, the present study provides new scientific evidence on the psychometric properties of the DASS-21 in young Peruvians in the university setting, which ratifies it as a brief, simple, and representative measure for the evaluation of mental health from a psychopathological approach. The scale allows for the identification of signs and symptoms of three highly prevalent disorders, which can manifest in an isolated or simultaneous manner. This condition favors the execution of timely care interventions and the planning of preventive and promotional actions in mental health with its consequent evaluation of effectiveness.

The results also have methodological implications, by promoting future research aimed at evaluating the sensitivity and specificity of the scale against instruments with proven diagnostic validity in anxiety, depression, and stress, in order to optimize its quality as a screening tool, in accordance with the requirements of the International Test Commission.

In conclusion, the DASS-21 shows evidence of validity and reliability, as well as invariance according to sex in the evaluated university student sample
