# Supplementary material for: Psychometric evaluation of the Depression, Anxiety and Stress Scale (DASS-21) in peruvian university students
Source: Rev Peru Med Exp Salud Publica. 2025 Sep 29;42(3):291–9. doi: 10.17843/rpmesp.2025.423.14209 (PMC12679971; doi:10.17843/rpmesp.2025.423.14209)
Supplement: Translation

# Translation: Evaluación psicométrica de la escala de Ansiedad, Depresión y Estrés (DASS-21) en estudiantes universitarios peruanos

**Authors:** Ingrid Cirilo-Acero, Marivel Aguirre-Morales, Josué Michael Franco-Mendoza, Lizley Janne Tantalean-Terrones, José Livia-Segovia

**Affiliations:** 1 Facultad de Psicología, Universidad Nacional Federico Villarreal, Lima, Perú.

**Keywords:** Estudios de Validación, Psicometría, Estudiantes Universitarios, Ansiedad, Depresión, Estrés psicológico

## Abstract

**Objetivos.:**

Evaluar las características psicométricas de la escala de Depresión, Ansiedad y Estrés (DASS-21) en universitarios de Lima, Perú.

**Materiales y métodos.:**

Se realizó un estudio instrumental en 1163 estudiantes de una universidad estatal, donde se evaluó la validez y confiabilidad de la DASS-21, en concordancia con los estándares internacionales.

**Resultados.:**

Se efectuó el análisis factorial confirmatorio con un primer modelo de tres factores (x^2^=706,5; gl=186; CFI=0,986; TLI=0,985; RMSEA=0,049; SRMR=0,057) y otro de segundo orden (x^2^=706,5; gl=186; CFI=0,986; TLI=0,985; RMSEA=0,049; SRMR=0,057), en el cual se reportó medidas de ajuste esperados. Además, se estableció asociaciones con otras medidas, salud mental positiva (r>-0,49) cuyos resultados fueron correlaciones inversas y valoraciones moderadas, estadísticamente significativas. También, se calculó las medidas de la invarianza métrica en función al sexo. Los coeficientes de confiabilidad, omega, alfa ordinal y Gutman alcanzaron valores mayores a 0,90 según lo esperado.

**Conclusiones.:**

La DASS-21 evidenció validez del análisis de la estructura interna, validez de criterio concurrente, discriminante y fiabilidad por consistencia interna como también es invariante en función a hombres y mujeres.

## INTRODUCCIÓN

Los estudios epidemiológicos sobre la salud mental en el Perú durante las dos últimas décadas han evidenciado la desafiante realidad de la depresión, la ansiedad y el estrés. En Lima Metropolitana, dos estudios realizados en 2002 y 2012 reportaron que la prevalencia de vida de los trastornos de salud mental fue de 18,2% y 17,3% para episodios depresivos, y de 9,9% y 3% para el trastorno de ansiedad generalizada, respectivamente ^1^^,^^2^. En 2018, los centros hospitalarios y de salud registraron, en adultos de 18 a 65 años, una prevalencia de vida de 30,3% para trastornos depresivos, 7,9% para trastornos de ansiedad y 3,6% para trastorno de estrés postraumático, con mayor frecuencia en mujeres que en varones ^3^.

 La pandemia de la COVID-19 tuvo una considerable repercusión epidemiológica en la salud mental a nivel mundial, con énfasis en los trastornos de ansiedad, depresión y síntomas de estrés postraumático, identificados como los problemas más frecuentes en personas que se contagiaron con la enfermedad ^4^. En la población china, se reportó un impacto psicológico de moderado a grave, con prevalencias de 16,5% para síntomas depresivos, 28,8% para ansiedad y 8,1% para estrés ^5^.

 Asimismo, se ha evidenciado una mayor presencia de angustia psicológica en mujeres, así como puntuaciones más altas de estrés en personas de 18 a 30 años y en mayores de 60 años ^6^. En el Perú, el personal médico de la región de Arequipa presentó síntomas de depresión en 56,7% y de ansiedad en 35,7% ^7^. Del mismo modo, en un estudio de 64 493 residentes peruanos, 35% reportó síntomas asociados a episodios depresivos mayores; además, 59% de estos participantes refirió un diagnóstico previo relacionado con algún trastorno de salud mental ^8^.

Por tanto, resulta prioritario investigar la utilidad de instrumentos que evalúen los aspectos psicopatológicos y psicosociales mencionados, los cuales la literatura describe como constructos diferenciados para la depresión ^9^^-^^12^, la ansiedad ^13^ y el estrés ^14^, con el fin de favorecer el rigor metodológico y disponer de medidas actualizadas.

En este contexto, surgieron las escalas de Depresión, Ansiedad y Estrés (DASS), desarrolladas en Australia para evaluar tres estados emocionales negativos relacionados: depresión, ansiedad y estrés. Posteriormente, se diseñó una versión abreviada de 21 ítems ^15^^,^^16^, cuya principal ventaja radica en su carácter de autorreporte breve y de fácil aplicación ^17^. Desde el punto de vista empírico, la depresión se caracteriza por tristeza, disminución de la autoestima y pérdida de metas trascendentes; la ansiedad se asocia a respuestas situacionales de miedo y síntomas físicos; mientras que el estrés se define como un «estado de excitación y tensión persistente con un umbral bajo para sentirse molesto o frustrado» ^15^.

 La escala de Depresión, Ansiedad y Estrés (DASS-21) posee una calidad moderada en la evidencia de validez de contenido, alta calidad para la validez de estructura bifactorial y para la consistencia interna. En la validez de criterio, solo la subescala de depresión muestra suficiente evidencia de su alta calidad. La medida de la invariancia entre géneros muestra evidencia inconsistente de calidad moderada; mas no se presentan suficiente evidencia para indicar una baja calidad de fiabilidad de las subescalas. Estos datos corresponden a una revisión sistemática sobre 48 estudios psicométricos del instrumento ^18^.

En el contexto internacional, la DASS-21 se tradujo a diversos idiomas y sometió a análisis psicométricos que fortalecen el modelo estructural de tres factores. Es el caso de su evaluación en profesionales de la salud de origen persa, con puntajes (*x* ^2^/(df) = 1457/(186); p<0,001; RMSEA=0,078; TLI=0,906; CFI=0,917; SRMR=0,047) con confiabilidad test-retest (0,740-0,881; p<0,01) ^19^. También, una validez de constructo trifactorial en el rango de 0,837 a 0,863 en pacientes masculinos de nacionalidad malaya ^20^.

Sin embargo, en un estudio transcultural en universitarios de Brasil, Canadá, Hong Kong, Rumania, Taiwán, Turquía, Emiratos Árabes Unidos y los Estados Unidos se confirmó el modelo bifactorial, incluso la figura de tres factores (depresión, ansiedad y estrés) y un solo factor (malestar general) ^21^. Estas tres posibilidades de modelo se replican en universitarios y pacientes suecos con confiabilidad de 0,77 a 0,91 para las dimensiones de depresión ansiedad y estrés ^22^. Por otro lado, en una adaptación al idioma hindi aplicada a pacientes con diagnóstico de cáncer, se evaluó un modelo de cuatro factores; no obstante, el modelo de tres factores mostró mayor consistencia interna con alfa>0,990 ^23^.

En el Perú, la DASS-21 ha mostrado evidencia psicométrica en adolescentes del nivel básico regular, con un adecuado ajuste factorial y coeficientes alfa y omega superiores a 0,80, además de invarianza por sexo y edad ^24^. En población universitaria, se replicaron tanto la división factorial tripartita como la unidimensional, aunque se identificaron factores específicos de naturaleza residual ^25^. En usuarios de un centro de salud mental comunitario de Lima, se confirmó una estructura de tres factores en 12 ítems, con coeficientes de fiabilidad de omega de 0,83 para depresión; omega de 0,73 para ansiedad y omega de 0,71 para estrés ^26^.

La revisión de los antecedentes del instrumento evidencia la necesidad de contar con herramientas estandarizados para su uso en la práctica clínica y científica, que permitan un diagnóstico adecuado de los usuarios, la evaluación de la efectividad de las intervenciones y el desarrollo de estudios epidemiológicos de alto impacto ^17^. En este sentido, las evidencias psicométricas de la DASS-21 en el Perú requieren mayor exploración en población universitaria con la mayor cantidad posible de muestra cómo se sugiere en estudios previos ^25^. Asimismo, resulta relevante analizar las propiedades estructurales del instrumento para determinar si se mantienen entre hombres y mujeres, con el fin de valorar su capacidad discriminativa al tratarse de una herramienta de índole clínica.

Por lo expuesto, el objetivo de la investigación fue evaluar las características psicométricas de la DASS-21 en universitarios de una institución estatal de Lima. Los objetivos específicos fueron identificar la estructura interna, evaluar las evidencias de validez de criterio y analizar la consistencia interna de las puntuaciones.

**MENSAJE CLAVE**

Motivación para realizar el estudio. En Perú, los problemas de salud mental continúan en aumento, lo cual evidencia la necesidad de emplear un instrumento con estándares de validez y confiabilidad adaptado al contexto universitario peruano, que permita evaluar los constructos de depresión, ansiedad y estrés.

Principales hallazgos. Se demostró la validez de la estructura interna, la validez concurrente y la confiabilidad del instrumento en población universitaria.

Implicancias para la salud pública. La demostración de evidencia psicométrica respalda la continuidad de futuros estudios orientados a establecer la prevalencia de la depresión, la ansiedad y el estrés; además, permite su uso como prueba de tamizaje en el ámbito de la salud mental.

## MATERIALES Y MÉTODOS

### Diseño

La investigación se enmarcó en un diseño instrumental ^27^, ya que la DASS-21 fue evaluada en términos de validez y confiabilidad, en concordancia con los estándares internacionales.

### Población y muestra

En el estudio participaron 1163 de los 14 616 estudiantes matriculados en la Universidad Nacional Federico Villarreal (Lima, Perú). El tamaño de la muestra calculado considerando un margen de error del 3% y una variabilidad de proporción del 50% (p=0,5; q=0,5) ^(^^28^ requería al menos 995 participantes para un nivel de confianza del 95%. No obstante, previendo rechazos y pérdidas, se incluyeron 1163 estudiantes.

### Instrumentos

La DASS-21 es una versión abreviada del instrumento original elaborado por los autores Lovibond y Lovibond ^15^. Evalúa la ausencia, presencia e intensidad de síntomas emocionales subjetivos y específicos asociados a depresión y ansiedad; en el caso del estrés valora la activación frente a dificultades persistentes en la conducta. Cada escala consta de siete ítems, puntuados de 0 a 3, lo que determina un puntaje máximo de 21 por dimensión. Su aplicación se centra en un periodo temporal reciente. El instrumento fue elaborado originalmente en inglés y se adaptó al español a partir de 2002 ^29^.

La Escala de Salud Mental Positiva de Warwick-Edinburgh, desarrollada por Tennant *et al*. ^30^, se compone de 14 items de enfoque positivo, asociadas al bienestar emocional y funcional, con cinco alternativas de respuesta que valoran experiencias ocurridas en las dos semanas previas. Aunque inicialmente fue diseñada para monitorear a los participantes de un programa de promoción de la salud mental positiva, investigaciones posteriores resaltan su utilidad para detectar cambios clínicamente significativos. Este instrumento cuenta con evidencia psicométrica tanto en población universitaria como en población general ^30^. Presenta una estructura unifactorial de alta consistencia interna y adecuados indicadores de fiabilidad ^31^, además de haber sido contrastado en un contexto latinoamericano ^32^. Con estos antecedentes, se evaluaron sus propiedades psicométricas en una muestra similar a la del presente estudio, confirmándose su estructura unifactorial con índices de ajuste adecuados (CMIN=2,196; GFI=0,997; CFI=0,998; NNFI=0,998; RMSEA=0,043; SRMR=0,031), y una consistencia interna omega de McDonald y alfa ordinal de 0,826 y 0,799, respectivamente.

### Procedimiento y análisis de datos

La recolección de datos se realizó mediante un formulario virtual de Google, que incluyó la información sobre el objetivo del estudio, los instrumentos de evaluación, edad mínima (18 años), alcances de la participación y garantía de confidencialidad. El formulario contenía una sección de consentimiento informado; quienes lo aceptaban accedían a preguntas sobre sexo, edad, año de estudios y a 45 ítems correspondientes a las escalas seleccionadas. El tiempo promedio de respuesta fue de 10 a 15 minutos.

El reclutamiento se efectuó a través del envío del formulario al correo institucional de los estudiantes con matrícula vigente y mediante su publicación en la página oficial de Facebook de la universidad. Trascurrido 15 días, ante la necesidad de aumentar la participación, se amplió la difusión a grupos de WhatsApp estudiantiles. La fase de recolección duró 60 días. Finalizado el proceso, las respuestas se descargaron en un archivo Excel y posteriormente se analizaron con los paquetes estadísticos JASP (versión 0.16.1) y R (versión 4.1.2).

La evaluación psicométrica incluyó el cálculo de coeficientes de correlación producto momento de Pearson para estimar la capacidad discriminativa de los ítems ^33^^)^ y las relaciones entre dimensiones ^34^ mediante índices heterotrait-monotrait (HTMT) ^35^. Asimismo, se realizó el análisis factorial confirmatorio (AFC) para identificar las dimensiones del instrumento y calcular los coeficientes alfa ordinal y omega de McDonald para estimar la consistencia interna de las puntuaciones. Este proceso se realizó previa depuración de posibles valores extremos que generen sesgos de medición.

También se emplearon estimaciones de mínimos cuadrados ponderados diagonalizados ^36^, por ser el modelo más robusto frente a la heterocedasticidad, con soporte en características de mejora de la robustez de estimadores y un mejor manejo de la variabilidad del error al trabajar con la diagonalización de la matriz de varianza-covarianza. En tal sentido, se ejecutó el análisis con la emulación EQS del paquete estadístico libre JASP, en su versión 0.14.1.0, bajo un intervalo de confianza del 99% y un error de estimación del 5%.

El proceso implicó la estimación de los parámetros de ajuste de dos posibles modelos de medida de la DASS-21 a) trifactorial independiente y de un factor de segundo orden y b) trifactorial relacionado, al tomar en consideración la versatilidad que puede presentar el modelo estructural y de medida con bases nomológicas y discriminantes entre escalas ^37^^,^^38^.

Se consideró un criterio mínimo de índice de bondad de ajuste (GFI)>0,95, índices comparativos (CFI, TLI) y comparativos no normalizados (NNFI) ^39^, con valores mínimos superiores a 0,90 e índices de error esperados por debajo de 0,08, tanto para el error cuadrático medio de aproximación (RMSEA) la raíz cuadrática estandarizada del residuo (SRMR). Se empleó el diagrama de senderos (Path Diagram), con el modelamiento de ecuaciones estructurales (SEM) cargas superiores esperadas mayores a 0,30 ^36^, asimismo, la variabilidad proporcionada por ítem, factor y las medidas de regresión y covarianza entre variables latentes.

También, se analizó la validez concurrente en base al contraste de los factores de la DASS-21 con la Escala de Salud Mental Positiva de Warwick-Edinburgh desde un aspecto divergente. Considerando los valores de los coeficientes como grande (>0,70), moderada (>0,50) o pequeña (>0,30) ^40^. Finalmente, se realizó la estimación de la confiabilidad con omega de McDonald, alfa ordinal y de dos mitades de Guttman, con puntajes referentes >0,70 y correlaciones ítem-test >0,30 por elemento en cada factor ^41^.

### Aspectos éticos

El estudio siguió las normas del Comité de Ética de la Unidad de Investigación, Innovación y Emprendimiento de la Facultad de Psicología que se expresó con la aprobación del Dictamen N°003, en conformidad con RR.N°077-2022-UNFV de la Universidad Nacional Federico Villarreal, los principios establecidos por el Colegio de Psicólogos del Perú, la Asociación Psicológica Americana y la declaración de Helsinki, incluyendo solo a los participantes que aceptaron ser parte del estudio dando su conformidad ante el consentimiento informado.

## RESULTADOS

Del total de participantes, el 30,5% (355) fueron hombres y el 69,4% (808) mujeres. La edad de los estudiantes oscilo entre 18 y 32 años, con un promedio de 25 años y una desviación estándar de 3,72.

### Evidencias de validez por análisis factorial confirmatorio

Se procedió al análisis del modelo de medida de la Escala DASS-21, considerando la conformación de tres factores relacionados y tres independientes con un factor de segundo orden. El contraste de las medidas de ajuste frente al representativo de las puntuaciones por la media se llevó a cabo con la estimación robusta DWLS (Diagonally Weighted Least Squares) en la emulación EQS. Los resultados del análisis en estos dos modelos se muestran en la tabla 1.

**Tabla 1:** Índices de ajuste de la Escala de Depresión, Ansiedad y Estrés (DASS-21).

Modelo	Ajuste				Comparativos			Residuales	
	x^2^	gl	p	GFI	CFI	NNFI	TLI	RMSEA	SRMR
Tres factores	706,5	186	1,00	0,987	0,986	0,985	0,985	0,049	0,059
Segundo orden	706,5	186	1,00	0,987	0,986	0,985	0,985	0,049	0,059

x^2^: Chi cuadrado, gl: grados de libertad, p: nivel de significancia, GFI: índice de bondad de ajuste, CFI: índice de ajuste comparativo, NNFI: índice comparativo no normalizado, TLI: Índice de Tucker-Lewis, RMSEA: error cuadrático medio de aproximación, SRMR: raíz cuadrática estandarizada del residuo

Se observa que las medidas obtuvieron un ajuste adecuado en ambos modelos, siendo similares, superando los mínimos estándares establecidos (x^2^=706,5; gl=186; p<0,001; GFI=0,996; CFI=0,986; NNFI=0,985; TLI=0,985; RMSEA=0,049; SRMR=0,057). Gráficamente, el modelo es representado en la figura 1.

**Figura 1:** Diagrama de senderos que representa el modelo de primer orden de la DASS-21 con tres factores relacionados.

EST/e: estrés; ANS/ans: ansiedad; DEP/d: depresión

En el mismo sentido, cabe destacar que se obtuvieron medidas de invarianza paramétrica en función del sexo. Las variaciones entre los índices de ajuste comparativos y residuales por los criterios de invarianza fueron inferiores a 0,01, lo cual puede observarse en la tabla 2.

**Tabla 2:** Medidas de invarianza por sexo para el modelo de tres factores.

Medidas	x^2^	CFI	△CFI	RMSEA	△RMSEA	SRMR	△SRMR	p
Modelo	706,500	0,986		0,049		0,057		< 0,001
Configuracional	830,119	0,988	0,002	0,046	-0,003	0,063	0,006	< 0,001
Métrica	904,170	0,986	0,000	0,048	-0,001	0,066	0,009	< 0,001
Escalar	939,187	0,988	0,002	0,047	-0,002	0,064	0,007	< 0,001
Estricta	977,990	0,985	-0,001	0,047	-0,002	0,066	0,009	< 0,001

x^2^: Chi cuadrado, p: nivel de significancia, CFI: índice de ajuste comparativo, RMSEA: error cuadrático medio de aproximación, SRMR: raíz cuadrática estandarizada del residuo, △: variación

Las cargas factoriales obtenidas por la conformación del modelo se mantuvieron oscilantes entre 0,43 y 0,98 en todos los ítems con el modelo de factores relacionados, respetando la conformación original de la prueba. Las medidas de covarianza entre factores fueron de 0,846 entre depresión y ansiedad; de 0,877 entre depresión y estrés y de 0,984 entre ansiedad y estrés. Siendo así, se confirmó la conformación del modelo de medida de la DASS-21 con el modelo de primer orden y sus respectivas variables latentes, ratificando con la propuesta original sugerida en la literatura respecto a la escala, como se observa desde la tabla 3.

**Tabla 3:** Cargas factoriales y regresiones de variable latente del modelo.

Factor	Ítem	Estimación	EE	z	p	IC 95%		Carga
						Inf	Sup	
EST	es1	1,000	0,000			1,000	1,000	0,481
	es2	1,227	0,084	14,691	< 0,001	1,064	1,391	0,622
	es3	1,584	0,099	16,054	< 0,001	1,391	1,778	0,763
	es4	1,289	0,087	14,802	< 0,001	1,118	1,460	0,666
	es5	1,550	0,081	19,076	< 0,001	1,391	1,710	0,716
	es6	1,279	0,082	15,600	< 0,001	1,118	1,440	0,669
	es7	1,580	0,098	16,071	< 0,001	1,387	1,772	0,716
ANS	ans1	1,000	0,000			1,000	1,000	0,433
	ans2	1,157	0,086	13,409	< 0,001	0,988	1,326	0,591
	ans3	1,332	0,100	13,308	< 0,001	1,136	1,528	0,640
	ans4	1,679	0,119	14,057	< 0,001	1,445	1,913	0,741
	ans5	1,774	0,128	13,892	< 0,001	1,524	2,025	0,804
	ans6	1,603	0,125	12,819	< 0,001	1,358	1,848	0,713
	ans7	1,729	0,127	13,572	< 0,001	1,479	1,978	0,769
DEP	d1	1,000	0,000			1,000	1,000	0,743
	d2	0,851	0,040	21,225	< 0,001	0,772	0,930	0,597
	d3	0,885	0,042	21,294	< 0,001	0,804	0,967	0,698
	d4	1,195	0,046	26,238	< 0,001	1,106	1,284	0,853
	d5	1,012	0,041	24,467	< 0,001	0,931	1,093	0,732
	d6	1,044	0,048	21,895	< 0,001	0,950	1,137	0,768
	d7	0,947	0,044	21,773	< 0,001	0,862	1,033	0,728
DEP	ANS	0,846	0,015	57,373	< 0,001	0,817	0,875	0,846
DEP	EST	0,848	0,015	58,126	< 0,001	0,820	0,877	0,848
ANS	DEP	0,967	0,009	110,903	< 0,001	0,950	0,984	0,967

EST/es: estrés, ANS/ans: ansiedad, DEP/d: depresión, DAS: DASS-21, EE: error estándar, Z: estimación de carga en escala Z, p: nivel de significancia, IC: intervalo de confianza, Inf: inferior, Sup: superior.

La evaluación de validez divergente evidenció que salud mental positiva mostró relaciones moderadas con ansiedad (-0,488), depresión (-0,607) y estrés (-0,525). Respecto de la validez discriminante, mediante el HTMT ^35^^)^ cumplió con los umbrales establecidos, por debajo de 0,85-0,90 (según el criterio conservador) sugiere una buena validez discriminante, mientras que valores cercanos o superiores a 1 indican falta de distinción entre los constructos. Sin embargo, la relación entre ansiedad y depresión (0,852) se encontró en el límite del criterio de validez discriminante mientras que ansiedad y estrés (0,979) superaron el umbral de 0,90 lo que indica una posible falta de diferenciación entre estos constructos. No obstante, la relación entre depresión y estrés (0,852) estuvo en el límite superior aceptable como puede apreciarse en la tabla 4.

**Tabla 4:** Validez concurrente por correlaciones heterotrait-monotrait (HTMT).

	Ansiedad	Depresión	Estrés
Ansiedad	1,000		
Depresión	0,852	1,000	
Estrés	0,979	0,852	1,000

 Con relación al análisis de la varianza explicada (AVE) se observa que todos los valores están por encima de 0,50, lo que señala la validez convergente (tabla 5).

**Tabla 5:** Evidencias de confiabilidad por consistencia interna y varianza media extraída (AVE) de la Escala de Depresión, Ansiedad y Estrés (DASS-21).

Escala	N° ítems	Omega	Alfa	Guttman	AVE
Escala total	21	0,943	0,942	0,944	-
Estrés	7	0,849	0,845	0,848	0,533
Ansiedad	7	0,855	0,855	0,856	0,573
Depresión	7	0,891	0,892	0,894	0,676

AVE: varianza media extraída

### Medidas de confiabilidad

Los coeficientes de consistencia interna para las escalas de la DASS-21 reflejaron valores superiores a 0,80 para los estadísticos omega, alfa y Guttman que evidencian confiabilidad en las medidas obtenidas por la escala. Los resultados del proceso pueden apreciarse en la tabla 5, en donde se observan medidas entre 0,84 y 0,94.

Los resultados del análisis a la Escala DASS-21 contaron con evidencias de validez y confiabilidad para el uso de la escala en la población referida, confirmándose así tanto el modelo de medida como la estructura de la escala con los 21 ítems que conformaron la propuesta original en la literatura respecto de esta.

## DISCUSIÓN

La DASS-21 cuenta un con respaldo de evidencias psicométricas, tanto a nivel nacional como internacional, que confirman su estructura en diversos contextos ^24^^,^^26^. En este marco, la presente investigación tuvo como objetivo verificar las características psicométricas de la DASS-21 en una muestra de universitarios de Lima.

Mediante el AFC, se corroboró la validez del modelo trifactorial de la DASS-21, así como la pertinencia del modelo de segundo orden, con índices de ajuste acordes a los reportados en la versión original ^29^. En paralelo, se sostiene la estructura unidimensional del instrumento y la fortaleza de las variables de depresión, ansiedad y estrés como agentes independientes en muestras suecas ^22^, peruanas ^25^, y en participantes de Brasil, Canadá, Hong Kong, Rumanía, Taiwán, Turquía, Emiratos Árabes Unidos y Estados Unidos ^21^.

Diversos estudios como han evidenciado la adecuada calidad del funcionamiento de los ítems ^19^^,^^23^^,^^42^. También se ha demostrado que los modelos son invariantes respecto al sexo, lo que respalda su equidad en la medición entre hombres y mujeres con lo ya observado en población adolescente ^24^.

Respecto a las cargas factoriales de los ítems en cada escala de la DASS-21, se observó que las dimensiones de ansiedad y depresión alcanzaron valores cercanos a 0,70 lo que refleja el aporte de cada elemento en la explicación de la variabilidad de sus indicadores clínicos ^36^^,^^39^. En el mismo sentido, las cargas factoriales que miden el estrés presentaron en promedio menores magnitudes. Tienen una presencia importante en el modelo de tres factores y en el modelo de segundo orden.

Sobre el ajuste del modelo, como evidencia de validez de estructura interna mediante AFC, es necesario contrastar las ventajas del modelo original frente al modelo unifactorial de «malestar general» ^25^ con implicancias teóricas que permite la agrupación de indicadores ansiógenos, depresivos y de estrés, fuera de la perspectiva de Lovibond y Lovibond en función a cada constructo ^16^.

En la misma perspectiva, existen estudios con magnitudes muestrales similares que no coinciden con los resultados de la presente investigación al no demostrar la estructura tridimensional de la escala ^43^, como mayoritariamente se ha logrado en poblaciones y grupos etarios similares. Esto se atribuye a las diferencias culturales y simbólicas del lenguaje de las versiones de la escala desde su primera adaptación al español ^29^.

Para demostrar otra forma de evidencia de validez, se analizó la asociación con la Escala de Salud Mental Positiva. Los resultados mostraron correlaciones inversas; lo que indica que las puntuaciones de la DASS-21 en depresión, ansiedad y estrés corresponden a un constructo diferente al evaluado por dicha escala. Además, las correlaciones fueron moderadas y significativas en todos los elementos de contraste. Por lo tanto, las medidas de la DASS-21 evidenciaron la validez de criterio concurrente esperada según la hipótesis del estudio.

En cuanto a la confiabilidad por consistencia interna, es posible afirmar que las medidas de la DASS-21 alcanzaron valores óptimos superiores a 0,70, en múltiples ocasiones, lo cual es recomendado para indicadores derivados del análisis en función de los coeficientes alfa y omega. En el mismo sentido, se demuestra la calidad que los ítems sostienen a nivel de agrupamiento, pese a la ordinalidad de cuatro puntos, incluyendo al cero como opción de respuesta dentro de cada una de las escalas; cuestión a la que comúnmente se han asociado problemas de consistencia por el aporte de varianza de los elementos al momento de constituir el factor. Estos resultados son contrastables con los indicadores de 0,77 a 0,92, considerando para el modelo trifactorial del instrumento en personal de salud ^22^.

Una de las limitaciones del presente estudio fue emplear el muestreo no probabilístico. Sin embargo, el tamaño de muestra aplicada es aceptable para evidenciar la validez del instrumento ^44^^,^^45^. Los resultados no pueden generalizarse, ya que los participantes provinieron de una sola institución. Además, por ser un estudio transversal no fue posible estimar la estabilidad temporal mediante el test-retest. Tampoco se contó con muestras clínicas, lo que impidió analizar la validez discriminante y realizar estimaciones de especificidad y sensibilidad mediante curvas ROC, procedimientos necesarios para sustentar su aplicación diagnóstica.

Asimismo, el presente estudio aporta nueva evidencia científica sobre las propiedades psicométricas de la DASS-21 en jóvenes peruanos del ámbito universitario, que la ratifica como una medida breve, sencilla y representativa para la evaluación de la salud mental desde el enfoque psicopatológico. La escala permite identificar signos y síntomas de tres trastornos de alta prevalencia, las cuales pueden manifestarse de manera aislada o simultánea. Esta condición favorece la ejecución de intervenciones asistenciales oportunas y el planteamiento de acciones preventivas y promocionales en la salud mental con su consecuente evaluación de efectividad.

Los resultados también tienen implicancias metodológicas, al impulsar futuras investigaciones orientadas a evaluar la sensibilidad y especificidad de la escala frente a instrumentos con validez diagnostica comprobada en ansiedad, depresión y estrés, con el fin de optimizar su calidad como herramienta de tamizaje, conforme las exigencias de la Comisión Internacional de las Pruebas Psicológicas.

En conclusión, la DASS-21 muestra evidencias de validez y de confiabilidad, además de invarianza según sexo en la muestra de universitarios evaluada.
